# Harnessing deep learning for detection of diabetic retinopathy in geriatric group using optical coherence tomography angiography-OCTA: A promising approach

**DOI:** 10.1016/j.mex.2024.102910

**Published:** 2024-08-20

**Authors:** Pooja Bidwai, Shilpa Gite, Biswajeet Pradhan, Harshita Gupta, Abdullah Alamri

**Affiliations:** a Symbiosis Centre for Applied Artificial Intelligence (SCAAI) Symbiosis Institute of Technology, Symbiosis International (Deemed University) (SIU), Lavale, Pune 412115 India; b Symbiosis Institute of Technology, Symbiosis International (Deemed University) (SIU), Lavale, Pune 412115 India; c Centre for Advanced Modelling and Geospatial Information Systems (CAMGIS), School of Civil and Environmental Engineering, Faculty of Engineering & IT, University of Technology Sydney, NSW 2007, Australia; d Department of Geology and Geophysics, College of Science, King Saud University, Riyadh, Saudi Arabia

**Keywords:** Diabetic Retinopathy;, Geriatric population;, Optical coherence tomography angiography (OCTA);, Convolutional neural networks;, Deep learning;, Classification, Detection of Diabetic Retinopathy Using Deep Learning

## Abstract

The prevalence of diabetic retinopathy (DR) among the geriatric population poses significant challenges for early detection and management. Optical Coherence Tomography Angiography (OCTA) combined with Deep Learning presents a promising avenue for improving diagnostic accuracy in this vulnerable demographic. In this method, we propose an innovative approach utilizing OCTA images and Deep Learning algorithms to detect diabetic retinopathy in geriatric patients. We have collected 262 OCTA scans of 179 elderly individuals, both with and without diabetes, and trained a deep-learning model to classify retinopathy severity levels. Convolutional Neural Network (CNN) models: Inception V3, ResNet-50, ResNet50V2, VggNet-16, VggNet-19, DenseNet121, DenseNet201, EfficientNetV2B0, are trained to extract features and further classify them.

Here we demonstrate:

• The potential of OCTA and Deep Learning in enhancing geriatric eye care at the very initial stage.
• The importance of technological advancements in addressing age-related ocular diseases and providing reliable assistance to clinicians for DR classification.
• The efficacy of this approach in accurately identifying diabetic retinopathy stages, thereby facilitating timely interventions, and preventing vision loss in the elderly population.

## Graphical abstract

**Figure:**
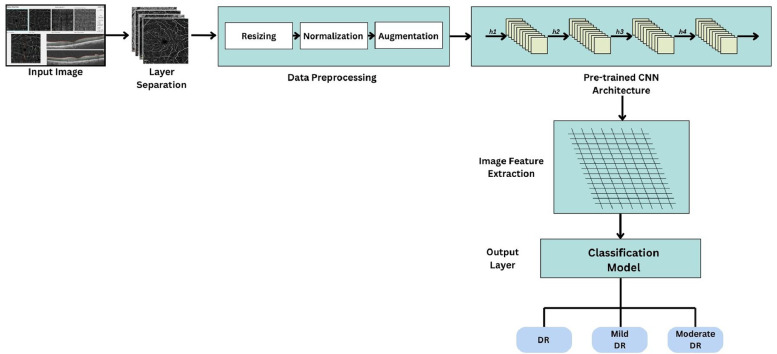

Specifications table

**Table:** 

Subject area:	Computer science
More specific subject area:	Image Processing.
Name of your method:	Detection of Diabetic Retinopathy Using Deep Learning
Name and reference of the original method:	N/A
Resource availability:	Optical Coherence Tomography Angiography-OCTA Dataset for Detection of Diabetic Retinopathy [Data set]. Zenodo. https://doi.org/10.5281/zenodo.10400092

## Background

Diabetic retinopathy (DR) is a complex disease involving microvascular damage, inflammation, and neurodegeneration in the retina, highlighting the need for innovative treatments and early detection methods [1]. Diagnostic tools for DR include ophthalmoscopy, fundus photography, fundus fluorescein angiography (FFA), optical coherence tomography (OCT), and optical coherence tomography angiography (OCTA), with FFA being the traditional gold standard [2]. However, OCTA offers a non-invasive alternative, utilizing motion contrast imaging to generate volumetric angiography images without the need for dye injection, presenting a safer option for patients [3]. Artificial intelligence (AI) is poised to revolutionize DR screening by enhancing accessibility, cost-effectiveness, and efficiency through telehealth and by improving the accuracy of retinal image analysis for early diagnosis [[4], [5], [6]]. In the realm of medical imaging and diagnostics, the classification of Diabetic Retinopathy (DR) has garnered significant attention, generating a wealth of research aimed at enhancing detection and grading methodologies. Notably, the application of Convolutional Neural Networks (CNN) has emerged as a cornerstone in this field, offering promising avenues for accurate and efficient diagnosis. Gondal et al. [7] proposed a CNN model for the identification of referable diabetic retinopathy (RDR), leveraging two publicly available fundus image datasets. Their approach involved a binary classification system, distinguishing between non-recommendable and recommendable DR, based on the severity of the condition. Training on the Kaggle dataset and testing on DiaretDB1, they achieved a sensitivity of 93.6% and a specificity of 97.6%, marking a significant stride in the automated detection of RDR. Building on this, S. Qummar et al. [8] explored the efficacy of an ensemble of five CNN models, including Inceptionv3, Resnet50, Xception, Densenet169, and Densenet121, on a Kaggle dataset comprising retinal images. This ensemble was engineered to harness the rich feature sets inherent in the data, aiming to elevate accuracy across all DR stages. Their findings underscored the potential of their model to outperform existing technologies, demonstrating superior detection capabilities of DR stages, and offering a substantial improvement over state-of-the-art methods utilizing the same dataset. Further innovations are evident in the work of researchers [9], who introduced a three-step program employing octal coherence tomography (OCT) for DR detection. This method focuses on segmenting retinal layers within OCT images, extracting 3-dimensional features that reflect both the first-order reflectivity and the thickness of the layers. Utilizing backpropagation neural networks for classification, they achieved an accuracy of 96.81% through leave-one-subject-out cross-validation, confirming the superiority of their method. In addition, the development of an automated Diabetic Retinopathy staging system using CNN for analyzing OCT images [10] marks a significant advance. This system not only outperformed traditional machine learning models but also surpassed human experts in accuracy and reliability for DR detection, achieving a quadratic weighted κ of 0.908 for the six-stage leveling task. Moreover, the use of a Multiple Instance Learning (MIL)-based CNN, specifically MIL-ResNet14, has shown promise for classifying DR in a weakly labeled dataset of widefield OCTA enface retina images [11]. This method demonstrated a remarkable resilience against adversarial attacks and high classification scores, highlighting its practical utility in clinical settings. The exploration of transfer learning for classifying OCTA images from a small dataset [12] and its application in a CNN model with the VGG16 architecture for DR detection using OCTA images [13] further illustrate the evolving landscape of DR diagnostics. These studies emphasize the potential of geometric data augmentation and transfer learning to enhance accuracy and model performance across various imaging devices, addressing critical research gaps such as the optimization of preprocessing techniques, the need for longitudinal studies, the interpretability of deep learning models, and tackling data imbalance and biases.

The development of this methodology for detecting diabetic retinopathy through Deep Learning applied to Optical Coherence Tomography Angiography (OCTA) images represents a significant advancement in the early diagnosis and management of this diabetes complication, a leading cause of blindness globally. It underscores a pivotal shift towards more efficient, scalable, and objective diagnostic practices, with the potential to significantly improve patient outcomes, reduce healthcare expenditures, and contribute to a deeper understanding of the pathophysiology of diabetic retinopathy.

## Method details

The methodology comprises a novel dataset obtained through a case-control study conducted among a geriatric population. It encompasses computational prerequisites, data pre-processing procedures, workflow delineation, CNN model specifications, feature extraction methodologies, and classification tasks. Each aspect is elaborated upon comprehensively in the following sections.

## Data description

Datasets for Diabetic Retinopathy detection pose various challenges since they require expert guidance and more patient cooperation to obtain optimal clarity of the image [14,15]. The primary dataset used in this study is the Optical Coherence Tomography Angiography (OCTA) dataset specifically developed for the detection of Diabetic Retinopathy. This dataset was carefully collected at Natasha Eye Care and Research Institute and reflects a concerted effort to harness advanced imaging technologies for ophthalmic research [16]. The dataset includes 262 high-resolution OCTA images representing a variety of retinal conditions across different stages of diabetic retinopathy.

## Characteristics of the dataset

Data Source Location: Natasha Eye Care and Research Institute, Pimple Saudagar, Pune.

Acquisition: Nonmydriatic OCTA images were acquired using the Optovue Avanti Edition machine. Images are classified into three categories: NO DR signs, Mild DR, and Moderate DR.

**Fig. 1:**
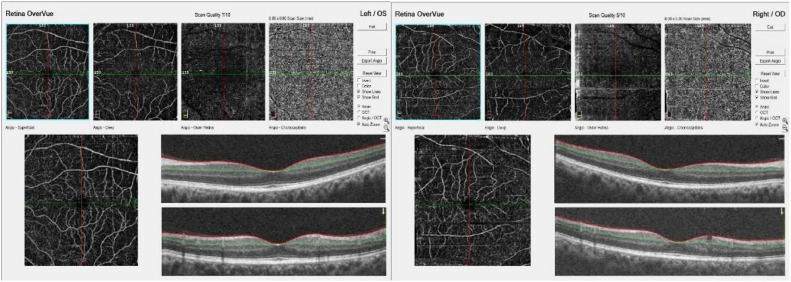
Sample OCTA images from the dataset.

Type of Data: High-resolution OCTA images, offering detailed views of the retinal vasculature, which are crucial for accurate detection and classification of diabetic retinopathy stages. OCTA (8 × 8 mm) images with dimensions 1596 × 990, 96 dpi, and jpeg. The dataset contains 262 OCTA images, carefully annotated, and verified by retina experts. Fig 1. Shows sample OCTA images from the dataset.

## Computational requirements

Hardware: Adequate computational resources are crucial for handling the processing demands of deep learning models and large datasets efficiently. A multi-core CPU is necessary for basic computation, while a GPU is highly recommended to significantly speed up the training and feature extraction processes involving deep learning models.

Memory: Sufficient RAM (at least 16GB, with 32GB or more being ideal) is required to manage large datasets and the operations of complex models in memory without significant slowdowns.

Software: The notebook is run in a Jupyter environment, which requires installation via Anaconda or directly through Python pip. Dependencies include TensorFlow, scikit-learn, pandas, and NumPy, among others.

## Data preprocessing

The data preparation phase is crucial for conditioning the images for optimal analysis by the CNN architectures. The raw OCTA images obtained, such as the one exemplified in Fig. 1 contain several layers of retinal structure that are relevant for the diagnosis and evaluation of Diabetic Retinopathy. Each of these layers offers unique information essential for the comprehensive analysis of retinal health. To prepare the images for feature extraction, the following preprocessing steps were applied to each image in the dataset:

### Layer separation

Initially, OCTA images are composed of different retinal layers, including the Angio-Superficial, Angio-Deep, Angio-Outer Retina, and Angio-Choriocapillaris, as well as cross-sectional optical coherence tomography angiography (OCTA) scans. The first step in preprocessing was to separate these layers. Each important layer was cropped from the raw image using a region of interest (ROI) approach. This step was performed carefully to ensure the capture of the entire vascular network and relevant anatomical features without introducing artifacts.

### Resizing

Once the layers were separated, each cropped image segment was resized to maintain a consistent input dimension across all images for the chosen CNN architectures. This standardization is vital to ensure that each model receives input of the same scale, which is crucial for comparative analysis.

### Normalization

Following resizing, the pixel values in each cropped layer were normalized. This normalization is not merely a scale adjustment to the range of 0 to 1 but also accounts for variations in imaging brightness and contrast that may occur due to different patient conditions or imaging equipment. The purpose of this step is to reduce model sensitivity to these inconsistencies, which are unrelated to the disease markers of interest.

### Augmentation

To address the limited size of the dataset and potential overfitting, data augmentation techniques were implemented. These included geometric transformations such as rotations and horizontal flips, as well as brightness and contrast adjustments. The augmentation was carefully calibrated to reflect realistic variations in OCTA imaging, enhancing the robustness of the subsequent feature extraction process.

### Quality assessment

Each image was subject to a quality assessment, as OCTA image quality can vary significantly due to patient movement or other factors during acquisition. Only images with a quality score above a predetermined threshold were included in the study to ensure reliable feature extraction. Images like the one provided, with a scan quality of 7/10, required careful consideration to determine if they met the quality criteria for inclusion.

### Final review

The processed images underwent a final review to ensure that all preprocessing steps were executed consistently and that the resulting images were ready for feature extraction. This review was an integral quality control step to prevent the introduction of biases in the comparative analysis of the CNN architectures.

By meticulously executing these preprocessing steps, we can ensure that the extracted features from each CNN architecture are truly representative of the underlying pathology and are not influenced by imaging artifacts or inconsistencies. This meticulous approach to data preparation is expected to yield more reliable and comparable results in our study.

## Workflow

### Import necessary libraries

Import libraries necessary for data manipulation, image processing, machine learning, and deep learning tasks. Key libraries include NumPy for numerical operations, pandas for data manipulation, TensorFlow for constructing and training deep learning models, and sci-kit-learn for various machine learning utilities.

### Setup CNN as feature extractor

Initialize a Convolutional Neural Network (CNN) model pre-trained on ImageNet data, such as VGG19. ConFig. the model to use its penultimate layer ('fc2′) as a feature extractor. This layer is chosen because it provides a robust and comprehensive representation of the image data, suitable for feature extraction.

### Data loading and feature extraction

Load image metadata from a CSV file that contains paths to images and their corresponding labels. Preprocess the images to fit the input requirements of the pre-trained CNN model. This involves resizing images to the required dimensions, converting them into arrays, and normalizing the pixel values to match the preprocessing used in the CNN training.

Pass the preprocessed images through the CNN to extract features. These features are then compiled into a dataset that is ready for use with machine learning models.

### Data splitting

Divide the dataset containing the extracted features and their corresponding labels into training and testing sets using a standard train-test split approach. The split is made in an 80%-20% ratio, with 80% of the data used for training the models and 20% reserved for testing and model evaluation. This split is crucial for training the models on a large subset of data while still retaining a separate subset for an unbiased evaluation of model performance.

### Classifier training and evaluation

Train two types of classifiers on the extracted features: a K-Nearest Neighbors (KNN) classifier and a simple feed-forward Neural Network. These models are chosen for their differing approaches to classification tasks, with KNN being a simple, distance-based algorithm and the Neural Network providing a more complex, nonlinear decision boundary.

Evaluate each classifier on the test set. Key performance metrics such as accuracy, precision, recall, and F1-score are calculated to assess each model's ability to accurately classify new, unseen images. This evaluation helps determine the effectiveness of the feature extraction and the overall utility of the trained classifiers.

## Overview of CNN architectures

### ResNet50V2

**Fig. 2:**
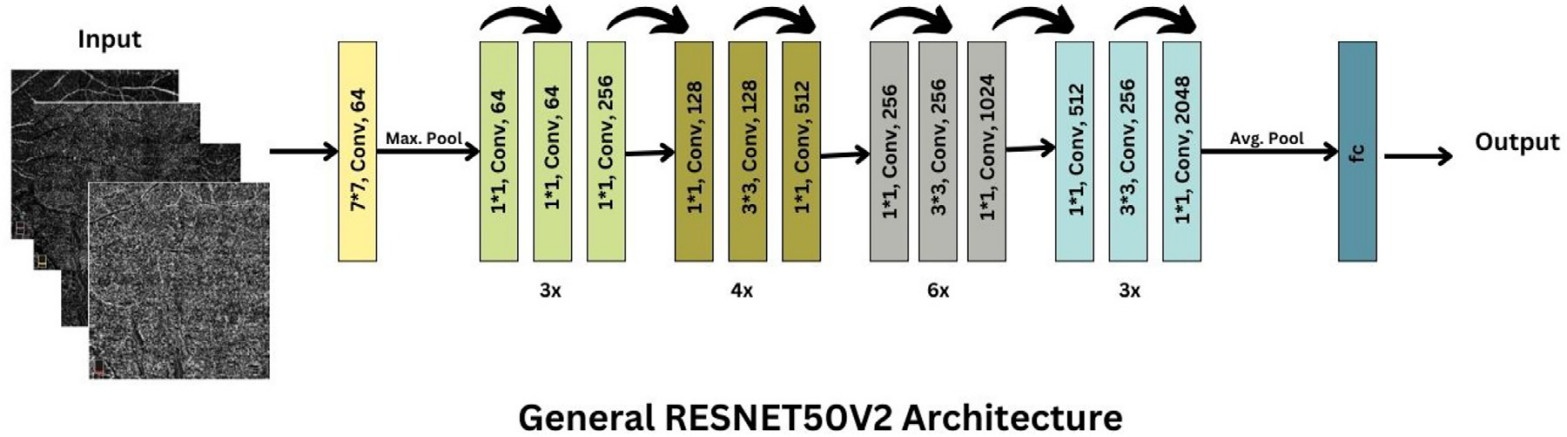
RESNET50V2.

In our study, we employed the ResNet50V2 architecture, known for its advanced residual learning framework with 50 layers featuring shortcut connections that prevent the vanishing gradient problem. Initially trained on the comprehensive ImageNet dataset, ResNet50V2 was adapted to diagnose diabetic retinopathy using OCTA retinal images. This adaptation involved using the 'avg_pool' layer for extracting essential global contextual information from the images, critical for identifying subtle indicators of the disease such as microvascular changes and hemorrhages. The model's capability to handle deep networks efficiently through its residual blocks enabled it to learn discriminative features necessary for medical diagnosis (Fig. 2).

## EfficientNetV2B0

In our study, we utilized EfficientNetV2B0, a model renowned for its scalability and efficiency in handling a range of different image resolutions, adapting seamlessly to varying computational constraints. Initially trained on the extensive ImageNet dataset, EfficientNetV2B0 was adapted to specifically address the complex patterns characteristic of diabetic retinopathy in OCTA retinal images. This model is distinguished by its compound scaling method, which uniformly scales the network width, depth, and resolution with a set of fixed scaling coefficients [17].

The EfficientNetV2B0 model was particularly effective due to its ability to process images at different resolutions while maintaining model efficiency.

### DenseNet121

**Fig. 3:**
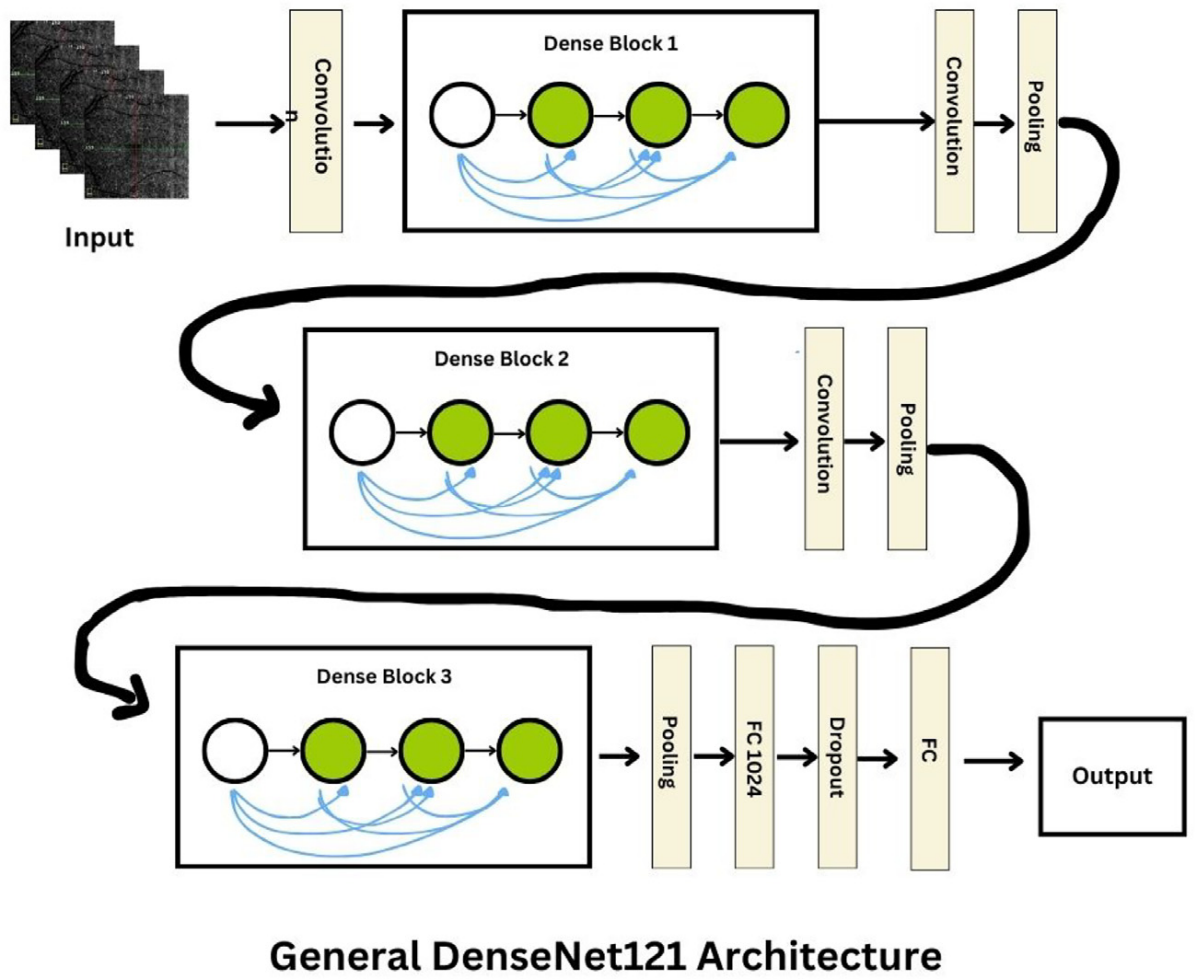
DenseNet121.

In our study, DenseNet121 was employed due to its distinctive architectural features that contribute to its efficiency in training and accuracy in performance. Known for its dense connectivity pattern [18], DenseNet121 connects each layer to every other layer in a feed-forward fashion, which significantly improves the network's ability to reuse features and reduces the problem of vanishing gradients. Initially trained on the ImageNet dataset, DenseNet121 was adapted for the specific task of classifying diabetic retinopathy using OCTA retinal images. This adaptation involved leveraging the model's feature-reusing capability to enhance the extraction of complex patterns indicative of the disease, such as microvascular changes and hemorrhages. The use of DenseNet121 in our diagnostic approach emphasizes its potential to improve the precision and reliability of medical imaging analyses, making it a valuable tool in clinical settings where diagnostic accuracy is crucial (Fig. 3).

### DenseNet201

**Fig. 4:**
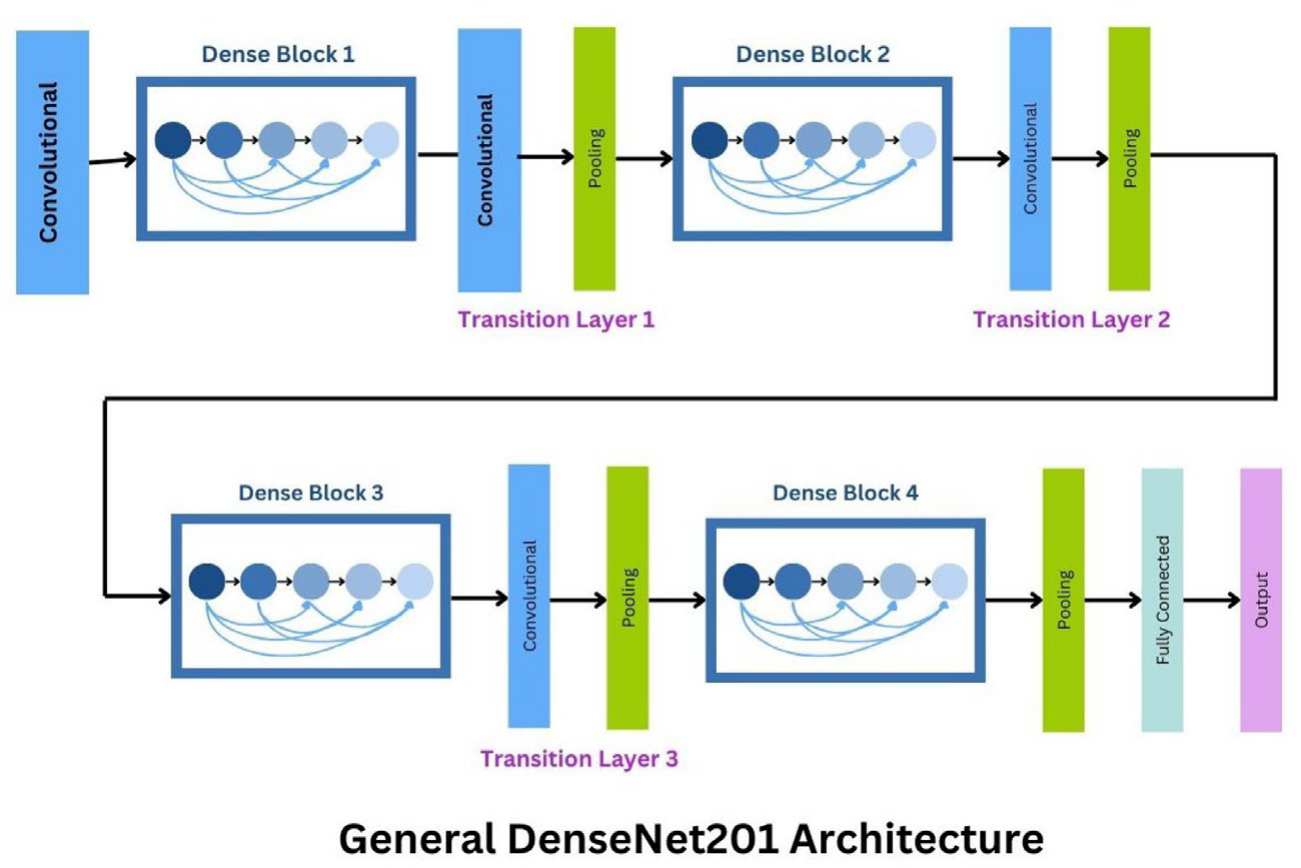
DenseNet201.

In our research, we implemented DenseNet201, a model celebrated for its unique architectural features that facilitate efficient feature utilization and gradient flow. DenseNet201, an extension of the DenseNet architecture, is characterized by its deeper structure and more layers, allowing for even more complex feature learning and reuse across the network. DenseNet201′s architecture employs a dense connectivity pattern where each layer is directly connected to every subsequent layer, ensuring maximum information flow between layers. This setup significantly reduces the vanishing gradient problem and enhances feature propagation, which is crucial for capturing the subtle nuances in medical images indicative of diabetic retinopathy, such as delicate vascular structures and early signs of hemorrhages (Fig. 4).

### Inception V3

**Fig. 5:**
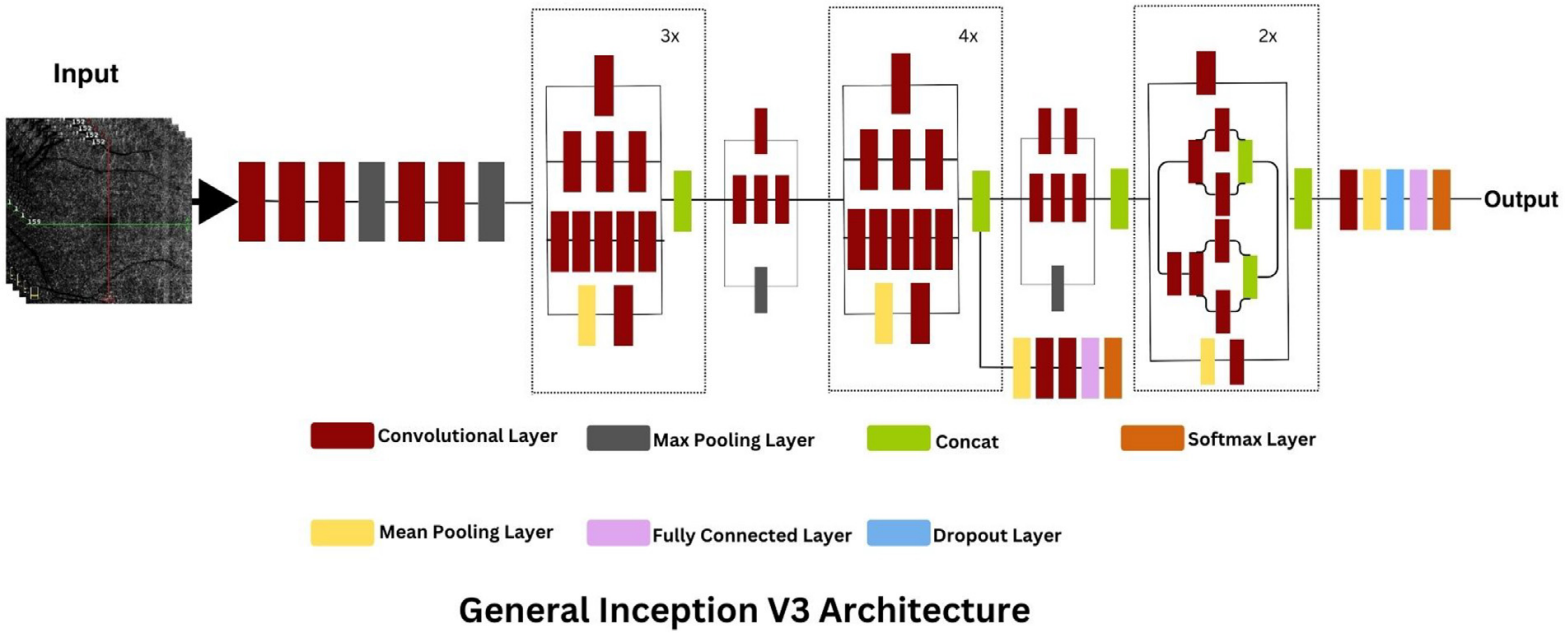
Inception V3 architecture.

It is a sophisticated convolutional neural network (CNN) that exemplifies innovation in network architecture design [19]. It is the third iteration of the inception family, characterized by its inception modules. These modules are smaller convolutions inside a larger convolution block, enabling the network to choose from various filter sizes (1 × 1, 3 × 3, 5 × 5) within the same layer. The inclusion of these parallel paths optimizes computational efficiency by allowing the model to adapt to the spatial hierarchies of features in images. This modular approach reduces the number of parameters compared to a densely connected network, minimizing computational load without compromising depth or breadth. For Diabetic Retinopathy detection, the varied granularity of feature extraction offered by Inception V3 can be particularly advantageous for capturing the intricate vascular changes characteristic of the disease (Fig. 5).

### VGG16

**Fig. 6:**
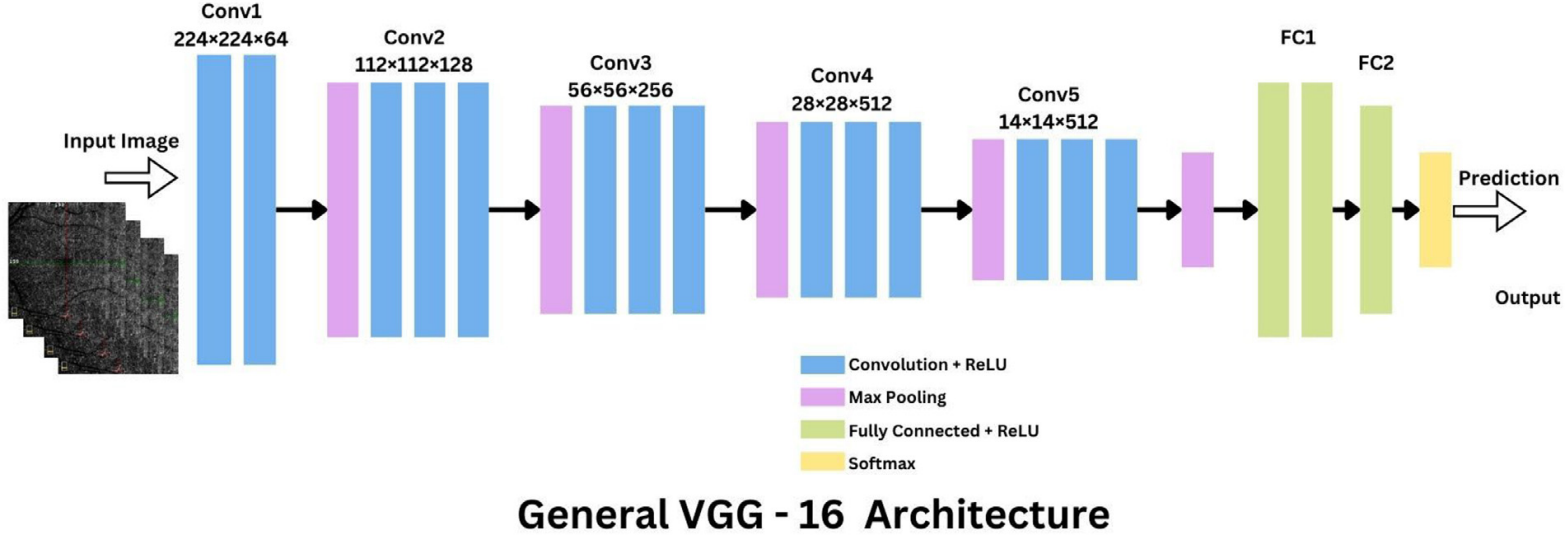
Representation of VGG16 architecture.

VGG16 is celebrated for its simplicity and uniformity, consisting of a series of convolutional layers followed by max-pooling layers, and fully connected layers towards the end [20]. The architecture uses small 3 × 3 receptive fields throughout the entire network, maintaining a fixed convolution stride, which is effective in capturing image features. The depth of the network, with 16 weight layers, allows it to learn from a large amount of data. However, this simplicity also results in a considerable number of parameters, which may lead to intensive computational demands. In the context of OCTA images, VGG16′s ability to capture fine-grained details through its uniform convolutions could be particularly useful for highlighting features such as microaneurysms or hemorrhages that are critical for accurate diagnosis (Fig. 6).

## Feature extraction process

The pre-trained models used, including VGG16, Inception V3, ResNet50 V2, EfficientNetV2 B0, DenseNet121, and DenseNet201, are equipped with weights from extensive training on the ImageNet dataset, which captures a broad spectrum of visual concepts and features. For efficient feature extraction from OCTA images, the classification layers of these networks were removed, allowing the core architectures to output detailed feature vectors. Specifically, features were extracted from the global average pooling layers in Inception V3 and ResNet50 V2 to reduce spatial dimensions while preserving important spatial hierarchies. In contrast, VGG19 utilized the last max-pooling layer, resulting in larger feature vectors due to the network's depth. EfficientNetV2 B0 adapted its depth for optimal feature scaling, and DenseNet121 and DenseNet201 leveraged their dense connectivity before transition layers to maintain information flow. This approach ensures that each model captures the most informative, higher-level abstract features necessary for accurately classifying stages of Diabetic Retinopathy, thus optimizing the discriminative capabilities of the extracted features.

## Classification tasks

The extracted features were fed into various classification algorithms. The choice of classifiers was guided by the need to evaluate the quality of the features in terms of their ability to distinguish between different classes in the OCTA dataset. Two main types of classifiers used are K-nearest neighbors (KNN) and Neural Networks.

KNN is a simple, non-parametric, and versatile algorithm used for classification and regression. It was chosen for its efficacy in pattern recognition which can be particularly beneficial when dealing with medical images where the distinction between classes may be subtle. The ease of understanding and implementing KNN, along with its capacity to make strong predictions with sufficient training data, made it a suitable choice for this study.

Neural Networks were used due to their ability to learn non-linear relationships and their adaptability in handling complex patterns in data. Their deep learning capabilities allow for a more nuanced understanding of medical images, which is essential for tasks such as identifying stages of Diabetic Retinopathy where features may not be linearly separable.

## Method validation

The performance of the classifiers, using the features extracted by the CNN architectures, was evaluated using a comprehensive set of metrics:

• Accuracy- Accuracy represents the proportion of true results (both true positives and true negatives) among the total number of cases examined [21]. High accuracy is essential in medical diagnostics to ensure reliable predictions for both the presence and absence of disease.
• Precision- Precision, or positive predictive value, measures the proportion of correctly identified positives out of all positive predictions made [22]. In the context of Diabetic Retinopathy, high precision would indicate that a high percentage of patients identified as having the condition truly have it.
• Recall (Sensitivity)- Recall, also known as sensitivity in medical diagnostics, measures the proportion of actual positives correctly identified [22]. It is crucial to ensure that the model identifies as many patients with the condition as possible.
• F1 Score- The F1 Score is the harmonic mean of precision and recall [22]. It is used when the balance between precision and recall is vital, which is often the case in medical diagnostics, as both false positives and false negatives carry significant consequences.
• AUC Score- The Area Under the Receiver Operating Characteristic curve (AUC-ROC) is a performance measurement for the classification tasks at various threshold settings [23]. The AUC Score provides an aggregate measure of performance across all possible classification thresholds.
• Kappa's Coefficient-Kappa's Coefficient measures inter-rater reliability for qualitative (categorical) items. It is used to assess the agreement between two raters on the assignment of categories [24]. In this study, it gauges the consistency of the classification results against a standard.
• Specificity- Specificity measures the proportion of actual negatives correctly identified (true negative rate) [25]. For Diabetic Retinopathy, high specificity means that healthy individuals are correctly identified as not having the disease, which is critical for avoiding unnecessary medical interventions.

Each metric offers a unique insight into the classifiers' performance and collectively provides a comprehensive evaluation of the models' effectiveness. The balance of these metrics is especially pertinent in medical applications, where the cost of false positives or negatives can be high, and the classifier's ability to generalize well and provide reliable diagnostics is paramount. By leveraging this suite of evaluation metrics, the study aims to present a detailed analysis of the classifiers' performance in Diabetic Retinopathy detection, offering a nuanced understanding of the strengths and weaknesses of the feature extraction capabilities of each CNN architecture (Table 1).

**Table 1:** Final validated results.

VGG19								
Model	Precision	Recall	F-score	AUC	Kappa	Accuracy N0_DR	Accuracy Mild_DR	Accuracy Moderate_DR
**Logistic Regression**	0.95	0.95	0.95	0.99	0.91	0.95	0.92	1
**SVM**	0.89	0.88	0.88	0.9	0.78	0.98	0.8	0.63
**Decision Tree**	0.66	0.64	0.65	0.71	0.39	0.67	0.6	0.62
**Random Forest**	0.88	0.87	0.86	0.97	0.75	0.98	0.8	0.5
**Gradient Boosting**	0.87	0.87	0.86	0.94	0.76	0.91	0.88	0.62
**KNN**	0.93	0.91	0.91	0.99	0.84	0.86	0.96	1
**Neural Network**	0.31	0.56	0.4	0.5	0.4	1	0.91	0.88
**ResNet50V2**								

**Model**	**Precision**	**Recall**	**F-score**	**AUC**	**Kappa**	**Accuracy N0_DR**	**Accuracy Mild_DR**	**Accuracy Moderate_DR**

**Logistic Regression**	0.87	0.87	0.87	0.97	0.77	0.86	0.84	1
**SVM**	0.84	0.83	0.82	0.86	0.69	0.86	0.88	0.5
**Decision Tree**	0.65	0.64	0.64	0.68	0.37	0.69	0.64	0.38
**Random Forest**	0.84	0.83	0.81	0.94	0.68	0.93	0.84	0.25
**Gradient Boosting**	0.9	0.89	0.89	0.95	0.8	0.95	0.88	0.63
**KNN**	0.94	0.93	0.93	0.99	0.88	0.93	0.96	0.88
**Neural Network**	0.92	0.92	0.92	0.98	0.86	0.93	0.88	1
**EfficientNetV2B0**								

**Model**	**Precision**	**Recall**	**F-score**	**AUC**	**Kappa**	**Accuracy N0_DR**	**Accuracy Mild_DR**	**Accuracy Moderate_DR**

**Logistic Regression**	0.92	0.92	0.92	0.99	0.86	0.91	0.92	1
**SVM**	0.9	0.89	0.89	0.9	0.81	0.91	0.92	0.75
**Decision Tree**	0.64	0.63	0.63	0.66	0.35	0.67	0.68	0.25
**Random Forest**	0.91	0.91	0.91	0.99	0.84	0.88	0.96	0.87
**Gradient Boosting**	0.93	0.93	0.93	0.98	0.88	0.93	0.96	0.87
**KNN**	0.9	0.84	0.85	1	0.74	0.71	1	1
**Neural Network**	0.92	0.92	0.92	0.99	0.86	0.91	0.92	1
**DenseNet121**								

**Model**	**Precision**	**Recall**	**F-score**	**AUC**	**Kappa**	**Accuracy N0_DR**	**Accuracy Mild_DR**	**Accuracy Moderate_DR**

**Logistic Regression**	0.95	0.95	0.95	0.99	0.91	0.95	0.92	1
**SVM**	0.9	0.89	0.89	0.89	0.8	0.98	0.88	0.5
**Decision Tree**	0.62	0.57	0.58	0.69	0.31	0.6	0.48	0.75
**Random Forest**	0.9	0.89	0.89	0.99	0.81	0.93	0.88	0.75
**Gradient Boosting**	0.92	0.89	0.9	0.99	0.82	0.81	1	1
**KNN**	0.94	0.92	0.92	1	0.86	0.86	1	1
**Neural Network**	0.91	0.91	0.91	0.99	0.83	0.91	0.92	0.88
**DenseNet201**								

**Model**	**Precision**	**Recall**	**F-score**	**AUC**	**Kappa**	**Accuracy N0_DR**	**Accuracy Mild_DR**	**Accuracy Moderate_DR**

**Logistic Regression**	0.96	0.96	0.96	1	0.93	0.98	0.92	1
**SVM**	0.93	0.92	0.92	0.92	0.85	1	0.88	0.63
**Decision Tree**	0.68	0.64	0.65	0.69	0.39	0.64	0.72	0.38
**Random Forest**	0.91	0.91	0.9	0.98	0.83	0.98	0.88	0.63
**Gradient Boosting**	0.95	0.95	0.95	0.99	0.9	0.98	0.92	0.88
**KNN**	0.9	0.89	0.89	0.98	0.82	0.86	0.92	1
**Neural Network**	0.98	0.97	0.97	1	0.95	1	0.92	1
**InceptionV3**								

**Model**	**Precision**	**Recall**	**F-score**	**AUC**	**Kappa**	**Accuracy N0_DR**	**Accuracy Mild_DR**	**Accuracy Moderate_DR**

**Logistic Regression**	0.92	0.91	0.91	1	0.84	0.86	0.96	1
**SVM**	0.89	0.88	0.88	0.88	0.78	0.91	0.92	0.63
**Decision Tree**	0.73	0.68	0.7	0.72	0.47	0.71	0.72	0.38
**Random Forest**	0.91	0.91	0.9	0.98	0.83	0.93	0.96	0.63
**Gradient Boosting**	0.89	0.88	0.88	0.98	0.79	0.86	0.96	0.75
**KNN**	0.91	0.88	0.88	0.99	0.8	0.79	1	1
**Neural Network**	0.93	0.92	0.92	0.99	0.86	0.88	0.96	1
**VGG16**								

**Model**	**Precision**	**Recall**	**F-score**	**AUC**	**Kappa**	**Accuracy N0_DR**	**Accuracy Mild_DR**	**Accuracy Moderate_DR**

**Logistic Regression**	0.91	0.91	0.91	0.99	0.84	0.91	0.92	0.88
**SVM**	0.9	0.89	0.89	0.89	0.8	0.98	0.88	0.5
**Decision Tree**	0.76	0.71	0.72	0.78	0.52	0.67	0.8	0.63
**Random Forest**	0.87	0.85	0.84	0.98	0.72	0.95	0.84	0.38
**Gradient Boosting**	0.9	0.89	0.89	0.98	0.81	0.93	0.88	0.75
**KNN**	0.95	0.95	0.95	0.99	0.91	0.95	0.96	0.88
**Neural Network**	0.88	0.88	0.88	0.99	0.79	0.88	0.88	0.88
**ResNet50**								

**Model**	**Precision**	**Recall**	**F-score**	**AUC**	**Kappa**	**Accuracy N0_DR**	**Accuracy Mild_DR**	**Accuracy Moderate_DR**

**Logistic Regression**	0.95	0.95	0.95	0.98	0.91	0.95	0.92	1
**SVM**	0.89	0.88	0.88	0.87	0.78	0.95	0.84	0.63
**Decision Tree**	0.76	0.75	0.75	0.77	0.56	0.81	0.72	0.5
**Random Forest**	0.9	0.89	0.89	0.96	0.8	1	0.8	0.63
**Gradient Boosting**	0.87	0.87	0.86	0.97	0.75	0.93	0.84	0.63
**KNN**	0.93	0.92	0.92	0.99	0.86	0.88	0.96	1
**Neural Network**	0.91	0.91	0.91	0.99	0.83	0.93	0.88	0.88

## Discussion

In a comprehensive analysis comparing classifiers like Logistic Regression, Support Vector Machine, Decision Trees, Random Forest, Gradient Boosting, K-Nearest Neighbors (KNN) and Neural Networks (NN) across eight different architectures like ResNet50, ResNet50V2, EfficientNetV2B0, DenseNet121, DenseNet201, InceptionV3, VGG16, and VGG19 varied performance metrics were observed that highlight the strengths and weaknesses of each model depending on the architecture. KNN consistently demonstrated robust performance with high scores across the board, particularly in accuracy, precision, recall, and F-score metrics. For example, in the VGG16 architecture, KNN achieved its highest metrics with a precision of 0.951, recall of 0.947, and an F-score of 0.948, along with an AUC of 0.9866, showcasing its efficacy in accurately classifying images with high consistency.

On the other hand, Neural Networks showed a strong ability to separate classes effectively, as evidenced by consistently high AUC scores across all architectures. The NN model excelled particularly with the DenseNet201 architecture, achieving the highest weighted precision, recall, and F-score values of 0.97, 0.97, and 0.97, respectively, and an impressive AUC of 0.99. This indicates that Neural Networks can leverage the depth and complexity of certain architectures to achieve superior predictive accuracy and reliability.

The results also reveal some challenges. For instance, while KNN performed exceptionally well in terms of overall accuracy and reliability, its performance in EfficientNetV2B0 was relatively lower, with precision, recall, and F-scores around 0.9, 0.84, and 0.85, respectively. This suggests a potential mismatch between the model capabilities and the architectural features of EfficientNetV2B0. Similarly, Neural Networks, despite their high separability capabilities, showed varied effectiveness across different architectures, with lower performance metrics in some cases, like with VGG16 where it recorded the lowest precision, recall, and F-score around 0.88, 0.88, and 0.88, respectively.

This analysis underscores the importance of selecting the appropriate model and architecture combination based on the specific requirements and challenges of the task at hand. While KNN is generally more consistent and reliable for broad use cases, Neural Networks are particularly adept at handling complex and nuanced patterns, making them suitable for tasks that benefit from deep learning's robust feature extraction and classification capabilities.

## CRediT authorship contribution statement

**Pooja Bidwai:** Conceptualization, Methodology, Data curation, Writing – review & editing. **Shilpa Gite:** Visualization, Investigation, Supervision. **Biswajeet Pradhan:** Visualization, Resources, Writing – review & editing, Funding acquisition. **Harshita Gupta:** Software, Writing – original draft. **Abdullah Alamri:** Visualization, Writing – review & editing, Funding acquisition.

## Declaration of competing interest

The authors declare that they have no known competing financial interests or personal relationships that could have appeared to influence the work reported in this paper.

## Data Availability

Data will be made available on request.
